# Fourteen‐month‐old infants track the language comprehension of communicative partners

**DOI:** 10.1111/desc.12751

**Published:** 2018-10-10

**Authors:** Bálint Forgács, Eugenio Parise, Gergely Csibra, György Gergely, Lisa Jacquey, Judit Gervain

**Affiliations:** ^1^ Laboratoire Psychologie de la Perception (LPP) Université Paris Descartes Paris France; ^2^ Laboratoire Psychologie de la Perception (LPP) CNRS Paris France; ^3^ Department of Cognitive Psychology Eötvös Loránd University (ELTE) Budapest Hungary; ^4^ Hungarian Academy of Sciences Budapest Hungary; ^5^ Department of Psychology Lancaster University Lancaster UK; ^6^ Cognitive Development Center (CDC) Department of Cognitive Science Central European University (CEU) Budapest Hungary; ^7^ Department of Psychological Sciences, Birkbeck University of London London UK

**Keywords:** experimental pragmatics, false belief, language acquisition, N400, social cognition, Theory‐of‐Mind

## Abstract

Infants employ sophisticated mechanisms to acquire their first language, including some that rely on taking the perspective of adults as speakers or listeners. When do infants first show awareness of what other people understand? We tested 14‐month‐old infants in two experiments measuring event‐related potentials. In Experiment 1, we established that infants produce the N400 effect, a brain signature of semantic violations, in a live object naming paradigm in the presence of an adult observer. In Experiment 2, we induced false beliefs about the labeled objects in the adult observer to test whether infants keep track of the other person's comprehension. The results revealed that infants reacted to the semantic incongruity heard by the other as if they encountered it themselves: they exhibited an N400‐like response, even though labels were congruous from their perspective. This finding demonstrates that infants track the linguistic understanding of social partners.

1

**RESEARCH HIGHLIGHTS**

- Fourteen‐month‐olds show an N400 effect even when only an observer encounters semantic incongruity (an object mislabeled), themselves not.
- Infants exhibit similar neural markers when following the linguistic understanding of a social partner as they show when processing word meaning.
- Results suggest that infant Theory‐of‐Mind goes beyond the attribution of false beliefs about object location as it allows attribution of miscomprehension as well.

## 2 INTRODUCTION

In order to be able to appraise the communicative value of words and to recognize their meaning, it is important to assume that others understand them in the context of their beliefs. Language is not the only means for information transmission, but it is a particularly efficient way to change the mental states of social partners. If human communication is fundamentally inferential in nature (Sperber & Wilson, 1995), communicators should ordinarily rely on their Theory‐of‐Mind (ToM) in monitoring and guiding their interlocutors’ comprehension of linguistic utterances.

Some evidence suggests that, when evaluating others’ interpretation of utterances, people take into account the information available to their partners. Keeping the common ground of a conversation has been proposed to drive acknowledgements, turn taking, and continued attention (e.g., Clark & Brennan, 1991), while people also seem to be influenced by the visual perspective of a conversational partner in communicational situations (e.g., Keysar, Barr, Balin, & Brauner, 2000). Rueschemeyer, Gardner, and Stoner (2015) devised a “joint comprehension task”, during which they recorded the event‐related potentials (ERPs) of participants, who read sentences with congruent or incongruent endings on a computer screen together with a confederate. The N400 ERP component is a well‐established neurophysiological indicator of semantic comprehension, which is sensitive to semantic violations, such as incongruent ending of sentences (Kutas & Federmeier, 2011; Kutas & Hillyard, 1980). Intriguingly, Rueschemeyer et al. (2015) found that when adult participants’—or even adolescents’ (Westley, Kohút, & Rueschemeyer, 2017)—task was to monitor the comprehension of the confederate, they produced an increased N400 for sentence endings that were incongruent for the confederate only. Crucially, only the participant, but not the confederate, had access to additional contextual information played back on headphones. This finding indicates that judging others’ semantic evaluation of linguistic utterances relies on tracking and taking into account the interlocutors’ beliefs.

Infants seem to be particularly well equipped for learning about linguistic and nonverbal communication. Newborns are already sensitive to the abstract structure (Gervain, Macagno, Cogoi, Peña, & Mehler, 2008) and certain acoustic characteristics (Gervain & Mehler, 2010) of language. They exhibit specific sensitivity to ostensive signals (Csibra, 2010), such as eye gaze, infant‐directed speech, or being addressed by their name, as measured by electroencephalography (EEG) around 5 months of age (Parise & Csibra, 2013; Parise, Friederici, & Striano, 2010) and by functional near‐infrared spectroscopy (fNIRS) around 6 months of age (Grossmann, Parise, & Friederici, 2010; Lloyd‐Fox, Széplaki‐Köllőd, Yin, & Csibra, 2015). By 6 months, infants expect speech to transmit information between agents even about invisible entities, such as intentions or preferences (Vouloumanos, Martin, & Onishi, 2014), and they already possess a small receptive vocabulary (Bergelson & Aslin, 2017; Bergelson & Swingley, 2012). Thus, the communicative value of speech seems to be evident for infants well before they produce their first words.

The capacity to represent others’ beliefs, which is crucial to be able to engage in inferential communication (Sperber & Wilson, 1995), seems to be present during the first year of life (Baillargeon, Scott, & He, 2010), perhaps as early as 6 or 7 months of age (Kovács, Téglás, & Endress, 2010; Southgate & Vernetti, 2014). Therefore, basic elements of ToM, in particular the ability to track others’ perspective and mental states, can contribute to language acquisition, especially to lexical development. Shortly after the first birthday, language comprehension seems to flourish indeed: word learning becomes more efficient and flexible (Werker, Cohen, Lloyd, Casasola, & Stager, 1998), words map onto concepts (Yin & Csibra, 2015), and the vocabulary is extended to abstract words (Bergelson & Swingley, 2013). The N400 can also be elicited around this age, but initially only under specific conditions. For example, high word producer 12‐month‐olds show it, but low word producers do not (Friedrich & Friederici, 2010). Infants as young as 9‐month‐olds exhibit an N400 if they hear objects named by their own mother, but not by an experimenter (Parise & Csibra, 2012), or if they are exposed to extensive familiarization with word–object pairings (Junge, Cutler, & Hagoort, 2012). By 14 months of age the N400 can be evoked reliably without these additional aids (Friedrich & Friederici, 2005, 2008), suggesting that semantic comprehension abilities become less dependent on situational aspects. Finally, at the same age, there is recognition of at least some sort of “common ground” (Moll, Richter, Carpenter, & Tomasello, 2008) in social interactions. These developmental steps seem to be critical for effective inferential communication, allowing infants to start to take into account what others mean and understand.

The experiments reported here addressed the question whether 14‐month‐old infants take the perspective of a communicative partner when they evaluate linguistic utterances, in particular, labels applied to familiar objects. As infants show evidence of visual perspective taking from at least 8 months of age (Kampis, Parise, Csibra, & Kovács, 2015), track others’ beliefs about objects, and evaluate referential expressions semantically, they should be able to judge how a communicative partner with a different perspective would interpret object labels.

## 3 EXPERIMENT 1

This experiment aimed to establish whether infants would display an N400 effect in a live setting where another person is also present. Previous studies with infants have shown semantic incongruity effects using video presentations, but never in live communicative contexts. During a presentation of familiar objects in a puppet theater, we recorded the EEG of 14‐month‐old infants, while a hand pointed to and a voice named the objects in the presence of an adult Observer. We measured infants’ ERPs time‐locked to the onset of the word label, which was either congruent or incongruent with the object for both parties.

### 3.1 Method

#### 3.1.1 Participants

Eighteen full‐term, 14‐month‐old infants (mean age: 444 days; range: 428–457 days) participated in this experiment. They were all French monolinguals (hearing no more than 15% of any other languages according to parental report). The sample size—for both experiments—was determined based on previous studies of the authors and other researchers investigating infant N400 effects (Friedrich & Friederici, 2005, 2008, 2010; Junge et al., 2012; Parise & Csibra, 2012). An additional 68 infants were excluded from the final data analysis (21% inclusion rate). The main reason for exclusion was not meeting the criteria of having at least 10 artifact‐free trials per condition (Stahl, Parise, Hoehl, & Striano, 2010) (*n* = 30), primarily because of eye movements (e.g., switching gaze from the Observer to the object or the other way around during the measurement period), blinking, or body movements. Further reasons for exclusion were fussiness (*n* = 25), refusing the EEG cap (*n* = 3), experimenter error (*n* = 2), or providing too noisy ERPs (*n* = 8).^1^ Such an attrition rate is not unprecedented in the infant EEG literature (cf. Parise, Reid, Stets, & Striano, 2008). It can be attributed to natural infant behavior, such as visual exploration of the scene, and also to the length of the experiment: a single trial lasted about 20–25 s because of the elaborate live experimental procedure, and together with the necessary short breaks, an infant needed to stay attentive for about 25–35 min to produce a sufficient number of artifact‐free trials. We believe that participant exclusion was unbiased with respect to any stable individual differences that would be relevant to the capabilities studied here. An additional four infants were tested during the training of the assistants playing the role of the Observer; the first couple of experiments with each new assistant were considered practice sessions, the data of which were not included.

#### 3.1.2 Materials

We used the audio recording of 15 object labels in French and selected corresponding real‐world objects that we suspected that infants knew the labels for (e.g., bunny, cup, shoe, duck, etc.). These word–object pairs were adapted from Parise and Csibra (2012), and were confirmed via the French version of the CDI (Kern, 2007). Additionally, we recorded seven short utterances to get infants’ attention, such as “Look!”, “How interesting!”, “Listen!” etc., in French, which were played back when needed (see Sections 3.1.2 and 3.1.3 below). A female speaker's voice was recorded for all audio materials; she uttered the words and the sentences in a language‐appropriate infant‐directed intonation. (For further details see Materials—Data S1)

#### 3.1.3 Apparatus

Infants sat on their parent's lap in front of a small puppet theater. A research assistant, playing the role of the Observer, sat facing them on the other side of the theater stage, behind a 30‐cm‐tall mobile occluder halfway on the stage and a curtain that she could open and close. A second experimenter sat on the left side of the stage behind a curtain, hidden to the infant, manipulated the occluder, and placed and replaced objects according to instructions displayed on a screen not visible to the participants. We used E‐prime 2.08 software (Psychology Software Tools) to control sound playback and to provide EEG triggers at the onset of word labels. We also video recorded the infants using a camera to check during later analysis for eye blinks, head and body movements, and to monitor their attention trial by trial.

#### 3.1.4 Procedure

Before the experiment commenced, we explained the procedure to the parents and obtained their informed consent. Ethics approval was obtained from the Ethics Committee of Université Paris Descartes. Parents were instructed to look downwards to the floor throughout the experiment and not to interact with or talk to their children, unless they felt it was necessary to soothe them.

**Figure 1:**
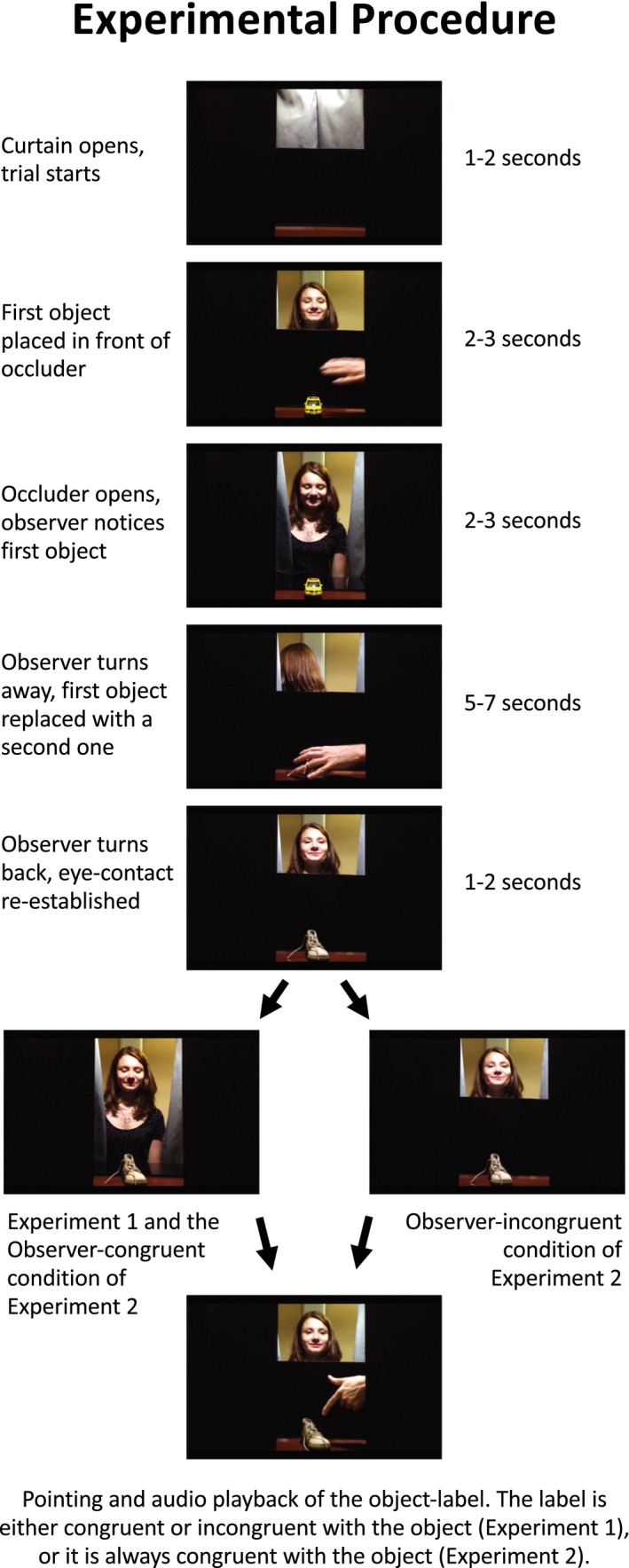
Experimental procedure. These events were performed in a live setting while the Observer was interacting with infant participants

In each trial (see Figure 1), the Observer first opened the curtains and established eye contact with the baby. An object (e.g., an apple) was placed in front of the occluder, so that the baby could see it while it was hidden from the Observer. Then the occluder was lowered so that the object was revealed to the Observer. She attempted to make eye contact again with the infant by looking up from the object. This gaze alternation was repeated until the Observer was certain that the infant knew that she had seen the object. If the infant was not attentive or fixated the object without looking at the Observer, she played back the pre‐recorded attention getting utterances using a hidden button box and, if necessary, manipulated the object briefly. The occluder was then raised, and the Observer turned 90° to the left so that her eyes were behind the curtain on the side, and the object was replaced with another object (e.g., a car) by the second experimenter from behind the curtain. When the second object was in place, the Observer turned back, and the second object was revealed to the Observer by lowering the occluder. (We included this change‐of‐object procedure in order to match the stimuli closely to the one to be used in Experiment 2.) The Observer again made sure that the infant noticed that she attended to the object. The second experimenter then raised the occluder again, pointed at the object from behind the curtain, and, using a hidden button box, the Observer played a recorded label that was, depending on the condition, either congruent or incongruent with the object. We took care to present the audio stimulus only when the infant was engaged. For 2 s following the playback, the Observer looked at the infant neutrally, while the pointing hand stood still, after which the object was removed, and the Observer closed the curtain. A 2s break indicated the end of the trial. The next trial started by opening the curtain. The order of objects and labels across trials was determined by a pseudo‐random sequence, in which the same object did not appear more than once in three consecutive trials, and there were no more than two congruent or incongruent trials in a row. A video recording of the procedure is provided in the Video S1.

#### 3.1.5 EEG recording and analysis

We recorded the EEG continuously via a 128‐channel Geodesic Sensor Net at 500 Hz sampling rate. Raw data were filtered with a 0.3 Hz high‐pass, and 30 Hz low‐pass filter, and segmented into 1,200 ms epochs starting at 200 ms before the onset of the audio playback in each trial. Artifact detection algorithms (e.g., for blinks, eye movements, bad channels, etc.) were used to exclude bad segments automatically, and video recordings confirmed that the infants attended the scenes in the retained segments. Infants must have had at least 10 artifact‐free segments per condition to be included in the final data analysis (Stahl et al., 2010). Bad channels were replaced by spherical spline interpolation, segments were averaged separately for the congruent and incongruent conditions, baseline‐corrected to the 200 ms preceding word onset, and re‐referenced to the average reference. Raw EEG data are available upon request. When relevant, Mauchly's test was used to test for sphericity violations, and whenever necessary the Greenhouse‐Geisser correction was applied. Statistical reports and effect size calculations follow the recommendations of Lakens (2013). (Further details are reported in EEG Recordings—Data S1).

### 3.2 Results

**Figure 2:**
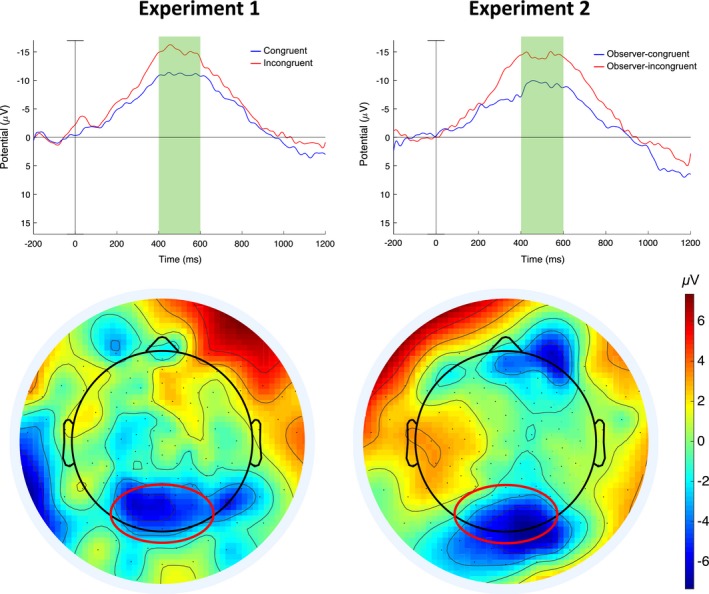
The N400 effect in Experiment 1 and Experiment 2. The upper panels show grand‐average waveforms over the parietal ROI. Negative is plotted up; time 0 is the onset of the audio playback. Green shadings indicate the time window of the infant N400 (400–600 ms). Topographical maps below show scalp distributions of ERP differences in the N400 time window. Colder colors indicate greater negativities. Red rings illustrate the ROI over which the N400 amplitude was quantified

Based on previous findings (Friedrich & Friederici, 2005, 2008, 2010), we identified a time window between 400 and 600 ms, and 13 parietal electrodes as the region of interest (ROI) for confirmatory statistical analyses. To quantify N400 activation, we computed the average amplitude within this time window and ROI. We found that the N400 was more negative in the incongruent (*M* = −13.8 μV, *SD* = 6.88) than in the congruent (*M* = −10 μV, *SD* = 6.86) condition, *t*(17) = −2.14, *p* = 0.047, 95% CI [−0.6 μV, −7.51 μV], Hedges's *g*_av_ = 0.53 (Figure 2). This confirmed that infants detected when labels did not match the object on the scene. Grand‐average topographical ERP plots for all electrode sites can be viewed in Figure S1.

### 3.3 Discussion

Our results demonstrate that 14‐month‐olds display signatures of semantic comprehension in a live setting, when attending object naming together with an adult. We found an N400 effect with a timing and distribution very similar to a typical infant N400.

## 4 EXPERIMENT 2

This study addressed our main question: whether infants take into consideration others’ mental states to follow their linguistic comprehension. We investigated whether 14‐month‐olds track word‐referent relations as understood by their communicative partners, and whether the underlying computations rely on the semantic content of object labels. We expected that an N400 effect would reflect infants’ evaluation of the semantic processing of object–label pairs from an observer's point of view.

In this experiment, the object name uttered was always congruent with the object from the perspective of the infant, but in half of the trials it was incongruent from the perspective of the Observer. We achieved this by inducing a false belief in the mind of the Observer about the identity of the object in the Observer‐incongruent condition (Figure 1). The Observer‐congruent condition was identical to the one of the first experiment, that is, the naming was congruent from both the Observer's and the infant's perspective.

### 4.1 Method

The methods of Experiment 2 were closely matched to those of Experiment 1 with the following differences.

#### 4.1.1 Participants

Eighteen full‐term, French monolingual 14‐month‐olds (mean age: 443 days; range: 425–456 days) participated in the experiment. An additional 62 infants were excluded from the final data analysis (23% inclusion rate). The main reasons for exclusion were as follows: not meeting the criteria of having at least 10 clean trials per condition (*n* = 27), fussiness (*n* = 17), refusing the EEG cap (*n* = 2), experimenter error (*n* = 4), or providing too noisy individual grand averages (*n* = 12). Further eight infants participated in the training sessions of the assistants.

#### 4.1.2 Procedure

In this experiment, the label was always congruent with the object on the scene, and the two conditions diverged from the point when the Observer turned back following the object change. In Observer‐congruent trials, the second object was revealed to the Observer by lowering the occluder, just as in Experiment 1. When the object was revealed to the Observer, both the participant and the Observer had a true belief about the identity of the object, and the label was congruent from both of their perspectives. In contrast, in Observer‐incongruent trials, the occluder was *not* lowered after the Observer turned back following the object change. The second experimenter pointed at the object from behind the curtain, and the Observer played back the recorded label of the second object. When the object was not revealed to the Observer, the naming was congruent with the object only for the infant but not for the Observer because she must have had no idea about the identity of the second object and falsely believed that the first object was labeled, from her perspective, incongruently.

### 4.2 Results

The amplitude of the N400 was analyzed the same way as in Experiment 1. We found significantly greater negativity to the Observer‐incongruent (*M* = −13 μV, *SD* = 7.51) as opposed to the Observer‐congruent (*M* = −8.42 μV, *SD* = 6.84) condition, *t*(17) = −2.46, *p* = 0.025, 95% CI [−0.65 μV, −8.55 μV], Hedges's *g*_av_ = 0.61 (Figure 2), indicating that the infants detected the incongruency for the Observer even if the labels they heard and the objects they saw were congruent with each other. To compare the findings of the two experiments we entered the data in a 2 × 2 ANOVA with Condition (congruent, incongruent) as within‐subject factor and Experiment (1 & 2) as between‐subject factor. The main effect of Condition was significant *F*(1, 34) = 10.6, *p* = 0.003, η_p_^2^ = 0.24, but neither the main effect of Experiment nor the interaction between Condition and Experiment was (all *p*s > 0.55).

**Figure 3:**
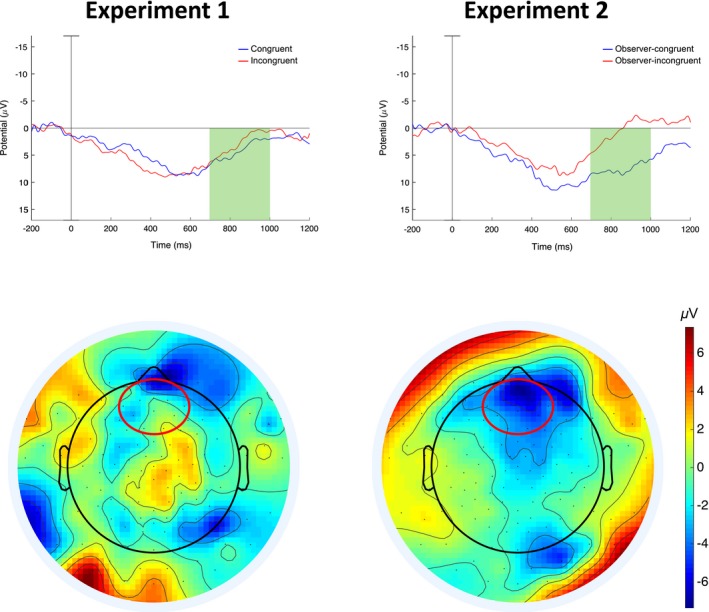
Frontal ERP effects in Experiment 1 and Experiment 2. Upper panels show grand‐average waveforms over the frontal ROI. Negative is plotted up; time 0 is the onset of the audio playback. Green shadings indicate where the frontal effect was significant (700–1,000 ms). Topographical maps below show ERP differences in the 700–1,000 ms time window for the two experiments. Colder colors indicate greater negativities. Red rings show the ROI over which the frontal negativity was quantified

Based on the visual inspection of the waveforms, we also identified a sustained frontal negativity starting from around 600–700 ms after stimulus onset (Figure 3). We ran exploratory statistical analyses over a frontal ROI consisting of 13 electrodes. We compared the mean amplitudes of 100 ms time windows between 600 and 1,000 ms after stimulus onset for the two conditions in a 2 × 4 ANOVA with Condition and Time Window as within‐subject factors. We found a significant main effect of Condition *F*(1, 17) = 9.75, *p* = 0.006, η_p_^2^ = 0.36 and of Time Window *F*(1, 51) = 15.5, *p* < 0.001, η_p_^2^ = 0.48, and a significant interaction of the two *F*(2, 34) = 7.34, *p* = 0.002, η_p_^2^ = 0.3. Planned comparisons revealed no difference between 600 and 700 ms, *t*(17) = −1.89, *p* = 0.076, Hedges's *g*_av_ = 0.49, but significant differences between 700 and 800 ms, *t*(17) = −2.71, *p* = 0.015, Hedges's *g*_av_ = 0.71; 800–900 ms, *t*(17) = −3.52, *p* = 0.003, 95% Hedges's *g*_av_ = 1.02; and 900–1,000 ms, *t*(17) = −3.51, *p* = 0.003, Hedges's *g*_av_ = 0.91 (Figure 3). Grand‐average topographical ERP plots for all electrode sites can be found in Figure S2 and further analyses in Results—Data S1.

### 4.3 Discussion

Our results demonstrate that 14‐month‐olds spontaneously evaluate the verbal understanding of communicative partners. The difference found between Observer‐congruent and Observer‐incongruent conditions indicates that infants track the mental states of social partners, keep such attributed representations updated, and use them to assess others’ semantic processing of words in relation to referents. Intriguingly, the timing and distribution of the N400 effect were very similar to what we found in Experiment 1, although the infants themselves should not have experienced any semantic incongruities between labels and objects. Such an incongruity must have originated from the semantic evaluation of the label–object match from the Observer's perspective, who must have believed that a different object was being labeled in Observer‐incongruent trials. Thus, our study provides indirect evidence for attributing false beliefs to the Observer.

The frontal effect, which we did not predict but found in Experiment 2, is comparable to previous findings regarding ToM by Liu, Sabbagh, Gehring, and Wellman (2009), although it is almost a second earlier than for preschoolers and is in the time window of adult mentalizing processes (Liu, Sabbagh, Gehring, & Wellman, 2004; Liu et al., 2009). In contrast to typical false belief tasks, the potentially communicative nature of our design and specifically the live setting we adopted could have induced online monitoring of beliefs of real partners, which could underlie infants’ adult‐like speed of mentalization. Our results also speak to the debate whether there are two ToM systems: an ancient yet efficient one available first and a flexible one developing later (Low, Apperly, Butterfill, & Rakoczy, 2016); or a single system, fully capable of attributing mental states early on (Baillargeon et al., 2010; Buttelmann, Suhrke, & Buttelmann, 2015; Kovács, 2016). Our finding, in particular the early frontal effect, suggests that similar systems could be at work in 14‐month‐olds as in adults. Moreover, implicit false belief tasks are often portrayed as capable of demonstrating false belief attribution about location, but not about identity, and it has been argued that only the latter would be evidence for the attribution of truly representational mental states (Low et al., 2016). Our results indicate that 14‐month‐old infants can attribute false beliefs about something that is not identity but is not location either: the existence of an object belonging to a specific basic‐level category at a certain location.

## 5 GENERAL DISCUSSION

The N400 ERP component is thought to reflect a violation of semantic expectancy (Brouwer, Fitz, & Hoeks, 2012; Kutas & Federmeier, 2011). Experiment 1 demonstrated that such an effect can be recorded from infants in a live setting when they match linguistic labels to objects. However, we also found this effect in Experiment 2, where no semantic violation was apparent from the perspective of the infants—as if the Observer's knowledge state was a constitutive part of the semantic context. Our finding therefore goes beyond the “social N400” effect reported by Rueschemeyer et al. (2015) and Westley et al. (2017): the explicit nature of their task could have invited top‐down control mechanisms to monitor semantic information available to the partner. In contrast, our task was not only implicit, as it was not possible to give instructions to infants, but the information asymmetry arose from differing perspectives, not from differential availability of semantic information. In other words, the infants did not need to assess the linguistic content of their social partner's belief, nevertheless, they spontaneously did so. Given the relatively high attrition rate, further research may be needed to replicate these results.

One potential source of explanation of this finding is that infants might rely on the same neural and cognitive mechanisms to accomplish a task for themselves as they do for others when processing mental states (Kampis et al., 2015). Consequently, infants could be utilizing comprehension mechanisms devoted to detecting semantic incongruities when they are motivated to guess how another person could have understood an utterance. A further potential explanation of our finding is that the N400 effect reflects a violation relative to the general, shared semantic context, a sort of common ground (Moll et al., 2008), or cognitive environment (Sperber & Wilson, 1995). Finally, it is also possible that, when they evaluate semantic relations, infants give priority to the communicators’ mental states (Southgate, 2014). The current experiment cannot sufficiently adjudicate among these accounts, but all these explanations share the requirement of tracking the semantic information available to communicative partners.

In sum, we found a neural marker of semantic incongruity detection in infants when their social partners, but not themselves heard objects labeled incorrectly. We also found a later frontal effect, similarly to studies investigating ToM, with a timing comparable to that of adults. Taken together, infants appear to be not only well prepared to learn language and track the mental states of social partners but also to appreciate language at its full potential in terms of inferential communication: as a system where words refer to things, have culturally fixed senses, and enable us to change each other's mental representations. Even before they start to speak, infants monitor the (mis)comprehension of communicative partners, raising the possibility that infants have a much more social take on language in general than previously thought.

## CONFLICT OF INTEREST STATEMENT

The authors have no conflict of interest to declare.

## Endnote

1 Individual ERPs were considered too noisy if they did not conform to a few classical indicators of successful recordings, such as: a flat pre‐stimulus baseline, initially aligned ERPs across conditions, and a mild positive deflection frontally and a mild negative deflection parietally, characteristic of auditory processing.
