# Supplementary material for: Fourteen‐month‐old infants track the language comprehension of communicative partners
Source: Dev Sci. 2018 Oct 10;22(2):e12751. doi: 10.1111/desc.12751 (PMC6492012; doi:10.1111/desc.12751)
Supplement: Supplementary file 1 [file DESC-22-na-s001.docx]

Fourteen-Month-Old Infants Track the Language Comprehension of Communicative Partners

Supplementary Material

# SI Procedure

The full list of labels that were presented to the infants is show below. One item from the set of Parise and Csibra (2012), “teddy” (“nounours” in French) was replaced with “nursing bottle” (“biberon” in French), because “nounours” is an ambiguous word in French, referring not only to teddy bear, but also to toy animals in general.

French words English translation

Balle Ball

Banane Banana

Biberon Nursing bottle

Canard Duck

Chaton Cat

Chaussette Socks

Chaussure Shoe

Chien Dog

Cuillère Spoon

Lapin Bunny

Livre Book

Pomme Apple

Tasse Cup

Téléphone Phone

Voiture Car

# SI EEG recordings

## EEG Acquisition

High-density continuous EEG was recorded using Hydrocel Geodesic Sensor Nets (Electrical Geodesics Inc., Eugene, OR, USA), with 124 or 128 scalp locations referenced to the vertex (Cz). The common ground electrode was at the posterior location (between Cz and Pz), standard for the net. The EEG signal was acquired using Electrical Geodesics Inc.’s Net Amps 400 amplifier, with a sampling rate of 500 Hz, using an anti-alias low-pass filter of 200 Hz.

## EEG data processing

EEG data were analyzed using Net Station 4.5.6 (Electrical Geodesics Inc., Eugene, OR, USA). A band-pass filter between 0.3-30 Hz was applied to the raw EEG signal offline. The signal was segmented into to two categories (Congruent and Incongruent in Experiment 1, Observer-congruent and Observer-incongruent in Experiment 2), where each ERP epoch started 200 ms before and 1200 ms after the onset of the auditory stimulus. Next, the following automatic artifact rejection algorithms were applied to the data: A channel was automatically marked bad and rejected if the difference between the minimum and maximum amplitude value within the segment, in an 80 ms moving window, exceeded 200 *μ*V at non-eye channels, 140 *μ*V at vertical eye channels, indicating blinks, and 55 *μ*V at horizontal eye channels, indicating horizontal eye-movements. The whole epoch was marked bad if more than 13 channels (10%) were marked bad. To ensure that infants paid attention at the time of playback, were engaged sufficiently to recognize the false belief of the Observer, and that the automatic algorithms picked up eye- and body-movements correctly, each trial was double checked using the video recording of the infants, and additional channels and segments were rejected manually if necessary. As a next step, bad channels were replaced using the interpolation algorithm of Net Station; then segments were averaged per condition; a baseline correction was applied using the 200 ms period preceding stimulus onset; and finally ERPs were re-referenced to the average reference.

Infants included in the final data analysis of Experiment 1 contributed, on average, 15.2 trials to the Congruent condition (SD = 5.6; range: 10-32), and 14.7 good trials to the Incongruent condition (SD = 4.31; range: 10-25). In Experiment 2, the average number of trials were 14.3 in the Observer-congruent condition (SD = 3.83; range: 10-22), and 14.2 in the Observer-incongruent condition (SD = 3.78; range: 10-21).

Based on previous findings (Friedrich & Friederici, 2005, 2008, 2010) and on visual inspection, the parietal region-of-interest (ROI) contained the following 13 electrodes: 62, 65, 66, 67, 70, 71, 72, 75, 76, 77, 83, 84, 90; while the frontal ROI the following 13 electrodes: 3, 4, 5, 9, 10, 11, 12, 15, 16, 18, 19, 22, 23.

# SI Results

To test whether the frontal negativity is specific to Experiment 2, we run a 2x2 ANOVA in the 700-1000 ms time window over the frontal ROI with Condition (congruent, incongruent) as within-subject and Experiment (1 & 2) as between-subject factor. The main effect of Condition was significant, *F*(1, 34) = 10.8, *p* = .002, *η*_p_^2^ = .24, with the Incongruent condition being more negative than the Congruent condition. Importantly however, there was a trending (Bonferroni-corrected) Condition x Experiment interaction as well, *F*(1, 34) = 5.18, *p* = .029, *η*_p_^2^ = .13, which was explained by planned comparisons showing a significant effect only in Experiment 2, *t*(17) = –3.42, *p* = .003, Hedges’s *g*_av_ = .92, but not in Experiment 1, *t*(17) = –.87, *p* = .4, Hedges’s *g*_av_ = .15.

# SI Figures

**Figure S1.** Grand-average ERP plots of Experiment 1 for all electrode sites. Negative is plotted upwards; frontal sites are above, parietal sites are below. Black line is the Congruent, red line is the Incongruent condition; green shapes indicate frontal and parietal ROIs. A typical infant N400 is apparent over posterior sites between 400-600 ms, but no frontal negativity can be observed.

**Figure S2.** Grand-average ERP plots of Experiment 2 for all electrode sites. Negative is plotted upwards; frontal sites are above, parietal sites are below. Black line is the Observer-congruent, red line is the Observer-incongruent condition; green shapes indicate frontal and parietal ROIs. An N400-like effect can be observed over parietal electrode sites between 400-600 ms, and a late negativity is apparent over frontal sites between 700-1000 ms.
